# In Vitro Cytopathogenic Activities of *Acanthamoeba* T3 and T4 Genotypes on HeLa Cell Monolayer

**DOI:** 10.3390/pathogens11121474

**Published:** 2022-12-05

**Authors:** Rosnani Hanim Mohd Hussain, Mohamed Kamel Abdul Ghani, Naveed Ahmed Khan, Ruqaiyyah Siddiqui, Shafiq Aazmi, Hasseri Halim, Tengku Shahrul Anuar

**Affiliations:** 1 Centre for Medical Laboratory Technology Studies, Faculty of Health Sciences, Puncak Alam Campus, Universiti Teknologi MARA, Bandar Puncak Alam 42300, Malaysia; hanim42.hussain@gmail.com; 2 Programme of Biomedical Sciences, School of Diagnostic and Applied Health Sciences, Universiti Kebangsaan Malaysia, Jalan Raja Muda Abdul Aziz, Kuala Lumpur 50300, Malaysia; profkamel@ukm.edu.my; 3 Department of Clinical Sciences, College of Medicine, University of Sharjah, Sharjah 27272, United Arab Emirates; nkhan@sharjah.ac.ae; 4 Department of Medical Biology, Faculty of Medicine, Istinye University, Istanbul 34010, Turkey; rsiddiqui@aus.edu; 5 College of Arts and Sciences, American University of Sharjah, Sharjah 26666, United Arab Emirates; 6 School of Biology, Faculty of Applied Sciences, Universiti Teknologi MARA, Shah Alam 40450, Malaysia; shafiqaazmi@uitm.edu.my; 7 Faculty of Pharmacy, Puncak Alam Campus, Universiti Teknologi MARA, Bandar Puncak Alam 42300, Malaysia; hasseri2945@uitm.edu.my; 8 Integrative Pharmacogenomics Institute, Puncak Alam Campus, Universiti Teknologi MARA, Bandar Puncak Alam 42300, Malaysia

**Keywords:** *Acanthamoeba*, cytopathic effect, HeLa cells, pathogenic potential, protease

## Abstract

Amoebic keratitis and encephalitis are mainly caused by free-living amoebae of the genus *Acanthamoeba*, which consists of both pathogenic and nonpathogenic species. The global distribution, amphizoic properties and the severity of the disease caused by *Acanthamoeba* species have inspired the scientific community to put more effort into the isolation of *Acanthamoeba*, besides exploring the direct and indirect parameters that could signify a pathogenic potential. Therefore, this study was performed to characterize the pathogenic potential of *Acanthamoeba* isolated from contact lens paraphernalia and water sources in Malaysia. Various methodologies were utilized to analyze the thermotolerance and osmotolerance, the secretion level of proteases and the cytopathic effect of trophozoites on the cell monolayer. In addition, the in vitro cytopathogenicity of these isolates was assessed using the LDH-release assay. A total of 14 *Acanthamoeba* isolates were classified as thermo- and osmotolerant and had presence of serine proteases with a molecular weight of 45–230 kDa. Four T4 genotypes isolated from contact lens paraphernalia recorded the presence of serine-type proteases of 107 kDa and 133 kDa. In contrast, all T3 genotypes isolated from environmental samples showed the presence of a 56 kDa proteolytic enzyme. Remarkably, eight T4 and a single T3 genotype isolates demonstrated a high adhesion percentage of greater than 90%. Moreover, the use of the HeLa cell monolayer showed that four T4 isolates and one T3 isolate achieved a cytopathic effect in the range of 44.9–59.4%, indicating an intermediate-to-high cytotoxicity level. Apart from that, the LDH-release assay revealed that three T4 isolates (CL5, CL54 and CL149) and one T3 isolate (SKA5-SK35) measured an exceptional toxicity level of higher than 40% compared to other isolates. In short, the presence of *Acanthamoeba* T3 and T4 genotypes with significant pathogenic potential in this study reiterates the essential need to reassess the functionality of other genotypes that were previously classified as nonpathogenic isolates in past research.

## 1. Introduction

*Acanthamoeba* species are the most extensive amphizoic protozoa in soil and aquatic environments in the world. They are free-living organisms and have been isolated from a diverse range of habitats, including freshwater lakes, seawater, swimming pools, hot tubs, air ducts in ventilation systems, contact lenses and in the atmosphere [1,2]. To date, several *Acanthamoeba* species are commonly known to cause sight-threatening amoebic keratitis (AK) among contact lens wearers, as well as acute granulomatous amoebic encephalitis (GAE) in immunocompromised individuals. According to Brindley et al. [3], 80% of the human population possesses the natural anti-*Acanthamoeba* antibody in their peripheral blood. Certain *Acanthamoeba* species could also cause various health complications, such as chronic sinusitis, cutaneous lesions and otitis in immunodeficient individuals, particularly HIV patients [4].

In addition, most of the *Acanthamoeba* isolates harbor numerous endosymbiont microorganisms, such as bacteria, yeasts, protists and viruses, which serve as important pathogens [5]. Khan [6] proposed that these pathogens can persist and proliferate within *Acanthamoeba* cells since they are protected from harsh environmental conditions and subsequently infect susceptible hosts. The widespread dispersion of *Acanthamoeba* in the natural environment is a major influential factor in *Acanthamoeba*-related diseases [7].

Given that the genus *Acanthamoeba* comprises both pathogenic and nonpathogenic species, their distinguishing features are crucial for clinical diagnosis. According to the currently applied morphological criteria, group II is classified as the most contagious *Acanthamoeba* species [6]. However, the available classification approach is unreliable at distinguishing between nonpathogenic and pathogenic species [8]. Interestingly, previous work on the full sequencing of nuclear small ribosomal subunit RNA genes *(Rns*) revealed that the genes consist of 12 highly variable regions that can be appropriately utilized as molecular tools to classify *Acanthamoeba* species [9]. Moreover, the *Acanthamoeba*-specific amplimer S1 (ASA.S1) has been detected in the *Rns* gene, which contains a diagnostic fragment of approximately 240 nucleotides, known as DF3, that is a highly variable and informative region for the genotypic characterization and identification of *Acanthamoeba*. Over the years, the ASA.S1-based polymerase chain reaction (PCR) and sequencing methods have been broadly employed for genotyping and diagnostic purposes [10]. Based on the *Rns* gene sequence, up to 22 *Acanthamoeba* genotypes (T1-T22) have been recognized so far with the preponderance of both *Acanthamoeba* infections being linked to the T4 genotype [11]. However, the relationship between pathogenicity and certain genotypes has yet to be established [12].

Furthermore, past studies have demonstrated that extracellular proteases of *Acanthamoeba* serve a vital function in pathogenic processes, including tissue invasion and cytolysis of host cells [13]. The primary step in AK involves the adhesion of the pathogens to host cells via glycoprotein–lectin interactions, followed by protease secretion and the cytopathic effect (CPE) [14]. Proteases are also heavily involved in the disruption of the blood–brain barrier in GAE [15]. Apart from predominantly producing serine proteases, *Acanthamoeba* isolates also secrete cysteine and metalloproteases with distinctly varying secretion patterns [6]. For example, several studies reported that clinical isolates produce a greater number of extracellular proteases compared to environmental isolates [16,17]. As such, Khan et al. [18] and Huang et al. [19] identified 107 kDa and 133 kDa serine proteases, respectively, which play a significant virulence factor in the pathogenic *Acanthamoeba*. Interestingly, *Acanthamoeba* isolates with strong pathogenic characteristics have developed a temperature tolerance of greater than 42 °C and concurrently display a high proteolytic enzyme activity [20].

T4 is the genotype most frequently associated with AK and GAE among the 22 identified *Acanthamoeba* genotypes [21]. Nevertheless, various isolated genotypes have been linked to clinical manifestations, including T2, T3, T5, T6, T11 and T15 [22,23]. Despite the fact that most protease characterization studies have evaluated strains that belong to the T4 genotype [24,25], the protease characterization from other genotypes is very limited. Therefore, this study aimed to conduct a comprehensive characterization of *Acanthamoeba* genotypes isolated from contact lens paraphernalia and environmental origins. The present study emphasizes the evaluation of the presence of virulence factors related to the pathogenic potential in *Acanthamoeba*, which includes the secretion of extracellular protease as well as in vitro cytopathogenicity. 

## 2. Materials and Methods

### 2.1. Isolation and Culturing of Acanthamoeba Samples

A total of 40 monoxenic isolates of *Acanthamoeba* were employed in this study, wherein 10 samples were collected from contact lens paraphernalia [10] and the remaining 30 samples were of distinct environmental origin [26,27]. Each sample was inoculated onto the center of a non-nutrient agar (NNA) plate (Sigma Aldrich, St. Louis, MO, USA), which consisted of Page’s amoeba saline (PAS) solution at pH 6.9 and preseeded with ultraviolet (UV)-inactivated *Escherichia coli* (strain K12, ATCC, Manassas, VA, USA) before being incubated at 30 °C and subjected to daily microscopic observation using an inverted microscope for up to 72 h [28]. In addition, cloned cultures were obtained from a single trophozoite or cyst using the dilution technique, as outlined by Costa et al. [29], which was then transferred to a 1.5% NNA plate to perform the physiological assays. Lastly, *Acanthamoeba castellanii* (ATCC 50492) was utilized as a positive control in this study. 

### 2.2. DNA Extraction and Genotype Isolation

The QIAamp^®^ DNA Mini Kit (Qiagen, Hilden, Germany) was used to extract DNA from the monoxenic *Acanthamoeba* culture following the manufacturer’s guidelines. Approximately, 10^4^ amoeba cells were transferred into Eppendorf tubes and centrifuged at 2,500 rpm for 10 min to obtain the pellet for the extraction process. The elution step was carried out using a final volume of 100 µL. The extracted DNA was then stored at −20 °C until further use.

Following the method by Schroeder et al. [30], the *Acanthamoeba*-genus-specific primers, namely forward JDP1 and reverse JDP2, were applied to conduct the PCR amplification of the approximately 450 bp fragment of the 18S rRNA region *Acanthamoeba*-specific amplimer ASA.S1 with similar cycling thermal conditions. Positive and negative controls were prepared by adding the extracted DNA of *A. castellanii* (ATCC 50492) and distilled water (replacing the DNA template) to the reaction mix, respectively.

The GenJET PCR Purification Kit (Thermo Fisher Scientific, Waltham, MA, USA) was employed to purify the PCR products, as per the manufacturer’s manual guidelines. Subsequently, a BigDye^®^ Terminator v.3.1 Cycle Sequencing Kit (Thermo Fisher Scientific, USA) was used to perform the sequencing analysis in both directions. Afterward, the MEGA software application, v.6.0.6 (Mega Software, Tempe, AZ, USA) was applied to analyze and edit the nucleotide sequence [31].

### 2.3. Physiological Assays

Physiological assays of thermo- and osmotolerance were conducted in triplicate using the cultures grown on an NNA medium supplemented with *E. coli*, following the protocols mentioned by Khan et al. [8]. The daily proliferation of *Acanthamoeba* species was observed using an inverted microscope for up to 72 h. Based on the previously reported dataset, the samples were categorized as high pathogenic potential, low pathogenic potential or nonpathogenic [32].

### 2.4. Preparation of Acanthamoeba Trophozoite Lysate

Growth of trophozoites on NNA plates was observed daily and harvested on the third day of the subculture. Trophozoites were gently scraped off from the agar surface of at least three plates from each sample with sterile PAS solution. The suspension was then rinsed twice with cold PAS solution before adding gentamycin (100 g/mL) and then centrifuged at 3500 rpm for 10 min. The resulting pellets were lysed at 1.5 × 10^7^ to 2.5 × 10^7^ trophozoites/mL by adding 0.15 to 0.20% (*v*/*v*) Triton^®^ X-100 prepared in water with or without proteinase inhibitor, followed by 1 or 2 cycles of a rapid freezing and thawing process. The protein content was evaluated based on the method by Tawfeek et al. [12], with slight modification with bovine serum albumin (BSA) as the standard.

### 2.5. Determination and Characterization of Protease Secretion via the Zymography Assay

The extracellular proteolytic activities of *Acanthamoeba* were determined using zymographic assays, as previously described [33]. *Acanthamoeba* trophozoite lysates were utilized in this study. Prior to the assay, zymography was prepared on the 10% SDS-polyacrylamide gels copolymerized with gelatin (1%). In brief, the prepared sample buffer was mixed to the *Acanthamoeba* trophozoite lysate up to a final volume of 30 to 40 µL (equivalent to 30 µg protein) before applying the solution to the gels. After electrophoresis, the gels were soaked in 2.5% Triton^®^ X-100 (*w*/*v*) solution for 60 min, followed by overnight incubation at 37 °C in a developing buffer (50 mM Tris-HCl, pH 7.5, containing 10 mM CaCl_2_). Finally, the gels were rinsed and stained with Coomassie brilliant blue R-250 (Bio-Rad Laboratories, Hercules, CA, USA). The destaining of the gels was performed until clear and sharp regions were visible over the dark blue background, which indicates protease activity [12]. Next, the destained gels were transferred into a storage solution to terminate destaining process. Subsequently, the Gel Analyzer 19.1 (www.gelanalyzer.com, accessed on 18 October 2022, Istvan Lazar Jr & Istvan Lazar Sr, Hungary) documenting system software was used to assess the molecular weights of the proteins. Identical experimental procedures were repeated using the trophozoite lysates from all isolates and pretreated with 1 mM phenylmethylsulphonyl fluoride (PMSF, Nacalai Tesque, Inc., Kyoto, Japan), which is a serine protease inhibitor, for 30 min prior to the electrophoresis.

### 2.6. Determination of the In Vitro Effect of Acanthamoeba on Cell Culture

#### 2.6.1. HeLa (Henrietta Lacks) Epithelial Cell Lines and Culture Conditions

HeLa cells were obtained from the American Type Culture Collection (ATCC CCL-2) and cultured in Dulbecco’s Modified Eagle Medium (DMEM) (^©^Capricorn Scientific, Ebsdorfergrund, Germany) containing penicillin (100 u/mL), streptomycin (100 pg/mL) (HiMedia Laboratories Pvt. Ltd., Maharashtra, India) and 10% fetal bovine serum (^©^Capricorn Scientific, Ebsdorfergrund, Germany) [34]. The cells were preserved in a 5% CO_2_ incubator at 37 °C with 80% humidity. Once the existing media was removed, confluent flasks were trypsinized with 2 mL trypsin (^©^Capricorn Scientific, Ebsdorfergrund, Germany) and incubated in 24-well plates (NEST^®^, Woodbridge, VA, USA). The flasks were used for the cytotoxicity and cytopathogenicity assays after the development of a uniform monolayer and were verified through microscopic examination within 48 h.

#### 2.6.2. Adhesion Assay

The adhesion assay was conducted to determine the binding ability of the *Acanthamoeba* isolates to the HeLa cell lines [35]. Approximately 24 h prior to the assay, the HeLa cell lines were grown in the prepared confluent monolayer in 24-well plates (NEST^®^, USA). Next, *Acanthamoeba* trophozoites (2 × 10^5^ cells/well) were transferred into the plates and incubated in a 5% CO_2_ incubator at 37 °C. The unbound amoebae were removed by washing with 1× PBS after 1 h incubation and a hemocytometer was used to count and calculate the number of bound amoebae, as follows: No. of unbound amoeba/Total number of amoebae × 100 = % unbound amoebae. Conversely, the number of bound amoebae was deduced as follows: 100% − % unbound amoebae = % bound amoebae.

#### 2.6.3. Cytopathic Effect Assay

Cultures of HeLa cell lines (ATCC CCL-2) were used to determine the ability of *Acanthamoeba* isolates to cause in vitro cytopathic effects (CPEs) [36]. The cells were cultivated in DMEM supplemented with penicillin (100 u/mL), streptomycin (100 pg/mL) (HiMedia Laboratories Pvt. Ltd., Maharashtra, India) and 10% fetal bovine serum (^©^Capricorn Scientific, Germany) at 37 °C in 5% CO_2_ for 24 h. The assay was performed in quadruplicate with 2 × 10^5^ of *Acanthamoeba* trophozoites per well in 24-well plates (NEST^®^, USA) containing a confluent monolayer of cells with an amoebae/cell ratio of 1:2. Wells containing HeLa cells that remain separated from the trophozoites were used as control samples. After 24 h of incubation, crystal violet was added into the wells for staining purposes and the visible change was analyzed macroscopically. ImageJ was used to categorize the results based on the degree of monolayer destruction, as follows: no CPE (-), CPE with up to 10% monolayer destruction (+), CPE with 10–50% monolayer destruction (++) and CPE with 50–100% monolayer destruction (+++) [37]. The *A. castellanii* (ATCC 50492) strain was employed as a positive control of CPE.

#### 2.6.4. Host Cell In Vitro Cytopathogenicity Assay

The cytotoxicity assay was carried out following the method by Castro-Artavia et al. [36]. In general, 96-well plates (NEST^®^, USA) containing confluent HeLa monolayers in DMEM were incubated in a 5% CO_2_ incubator at 37 °C for 24 h. The cytotoxic effects were determined by collecting the supernatants after the incubation period and measuring the lactate dehydrogenase (LDH) release using the cytotoxicity detection kit (Roche Applied, Burgess Hill, West Sussex, U.K.). The percentage (%) of cytotoxicity was calculated as follows: Percentage (%) cytotoxicity = (Sample value − Control value)/(Total LDH release − Control value) × 100. The control values were obtained from the host cells incubated in DMEM alone, while the total LDH release was determined from HeLa treated with 2% Triton^®^ X-100 at 37 °C for 1 h. In this assay, the cell-supernatant-containing LDH catalyzes the conversion of lactate to pyruvate, forming NADH and H^+^. Subsequently, the catalyst (diaphorase, solution from kit) transfers H and H^+^ from NADH and H^+^ to the tetrazolium salt p-iodonitrotetrazolium violet (INT), which is reduced to formazan (dye) and the absorbance is read at 490 nm. Accordingly, a cytotoxicity level of less than 10% is considered nontoxic, while cytotoxicity levels of 10–25% are deemed as low-toxic. In contrast, intermediate toxicity is represented by a cytotoxicity level of 25–40%. Ultimately, cytotoxicity levels of greater than 40% are regarded as highly toxic [38].

## 3. Results 

### 3.1. Physiological Tolerance of Acanthamoeba Genotypes

The molecular analysis of the ASA.S1 fragment of the 18S rRNA gene showed that the isolated *Acanthamoeba* samples from contact lens paraphernalia and environmental origins belonged to the T3, T4, T5, T11, T15, T17, T18 and T20 genotypes, as shown in Table 1. The obtained sequences were subsequently submitted to GenBank with the accession numbers MH790980–MH791025, MN700306–MN700310 and MN700270–MN700305. The physiological traits of the amoeba isolate from the contact lens paraphernalia included the ability to grow at 37 °C and 0.5 M mannitol (except for CL177). In addition, five T4 isolates (CL5, CL54, CL118, CL126 and CL149) from the 10 contact lens paraphernalia isolates were characterized as highly pathogenic, given their ability to survive at high temperatures (42 °C) and osmolarity of 1 M mannitol. Furthermore, nine environmental isolates (T4 = 7; T3 = 1 and T15 = 1) recorded a strong response towards physical stimulants. Comparatively, the growth of *A. castellanii* (ATCC 50492), which was as the reference strain in this study, also persisted at 42 °C and 1 M mannitol.

### 3.2. Secretion of Active Serine Protease by Acanthamoeba Isolates 

**Figure 1:**
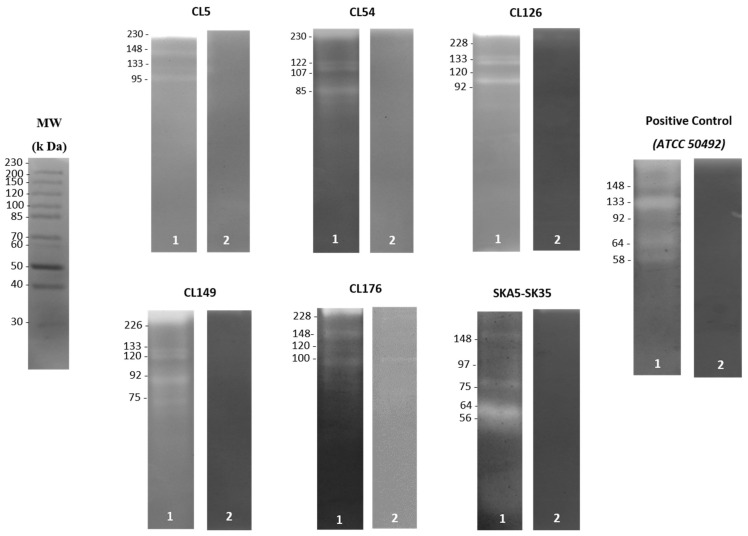
Zymography analysis of *Acanthamoeba* trophozoite lysate from contact lens paraphernalia and environmental isolates without protease inhibitor (lane 1) and pretreated with 1 mM PMSF (serine protease inhibitor) (lane 2). The molecular weight in kDa is indicated on the edge of each gel. Contact lens paraphernalia isolates: CL5, CL54, CL126, CL149 and CL176. Environmental isolate: SKA5-SK35.

In terms of the extracellular protease secretion, *Acanthamoeba* trophozoite lysate from all isolates demonstrated identical banding patterns on the gelatin gels. Besides the presence of serine proteases with higher molecular weights than 45 kDa, the zymography analyses showed that all isolates exhibited 1–7 protease bands without the protease inhibitor (Table 2). Moreover, a single T4 isolate (CL54) and three other isolates (CL5, CL126 and CL149) from the contact lens paraphernalia displayed the presence of protease patterns of 107 kDa and 133 kDa enzymes, respectively. On the contrary, the detected protease in most of the environmental isolates recorded a molecular weight range of 45–97 kDa, as depicted in Figure 1. The addition of PMSF (serine protease inhibitor) almost completely inhibited the protease activity of the trophozoite, except for several unclassified proteases with molecular weights of 80, 100, 152, 162 and 252 kDa, which were only partially inhibited. Thus, the results suggest varying secretion patterns of serine proteases in all isolates.

### 3.3. Adhesion of Acanthamoeba Trophozoite to HeLa Cell Monolayer

**Figure 2:**
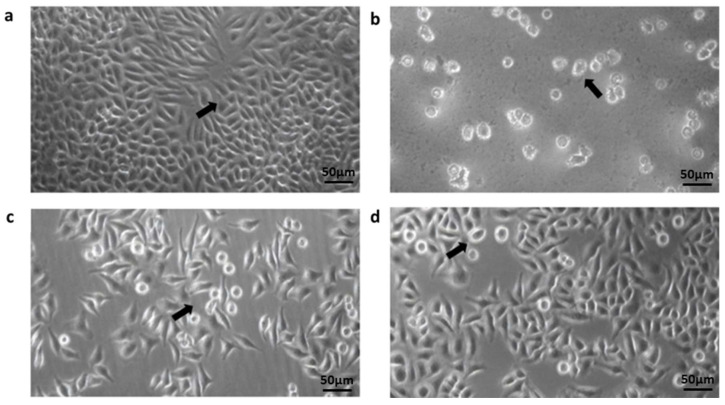
Inverted-light microscope image analysis of the interaction between HeLa cell line and *Acanthamoeba* trophozoites from the contact lens paraphernalia and environmental isolates. Images: The arrow shown in the images indicates: (**a**) The confluent monolayer appearance of the HeLa (ATCC CCL-2) cell line incubated for 24 h; (**b**) *Acanthamoeba* trophozoites adapting and attaching to the culture flask; (**c**,**d**) the coincubation between HeLa cell monolayer and trophozoites during the adhesion assays. Trophozoites were discovered in close proximity to the surface of epithelial cells as well as beneath the cell layer. Magnification in (**a**–**d**) was made at 20×.

The *Acanthamoeba* isolates were able to adhere to the HeLa cell monolayer at a percentage range of 57.5–98.5%. In addition, the adhesion assay revealed that eight T4 isolates (CL5, CL45, CL54, CL107, CL118, CL149, UTA4-HT14 and TB5-B27) and one T3 isolate (SKA5-SK35) strongly adhered to the cell lines with a high percentage of greater than 90% (Table 3). Conversely, four isolates (PD3-B12, TC4-B33, TC7-B34 and M8-B8) composed of genotypes T4, T11 and T18 exhibited less than 70% binding. Figure 2 presents the *Acanthamoeba* trophozoites that adhered to the morphological structure of the HeLa cell monolayer.

### 3.4. Cytopathic Effects of Acanthamoeba over HeLa Cell Monolayer

**Figure 3:**
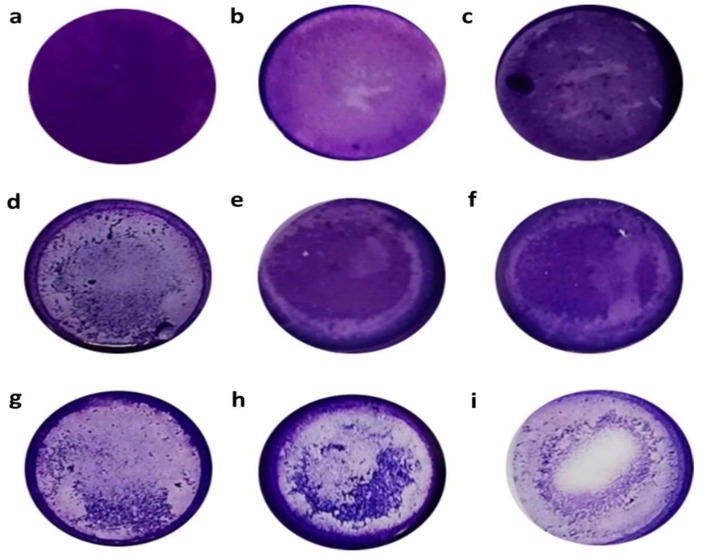
Crystal-violet stain that shows cytopathic effects (CPEs) of *Acanthamoeba* isolates over HeLa cell monolayer. Amoebae were incubated with HeLa cell line in 24-well plates for 24 h at 37 °C and their CPE was observed using the crystal-violet stain. Images: (**a**) HeLa cell control; (**b**,**c**) showing CPEs with up to 10% monolayer destruction; (**d**–**f**) represent CPEs with 10–50% monolayer destruction; (**g**,**h**) CPEs with 50–100% monolayer destruction; and (**i**) HeLa cells incubated with *Acanthamoeba castellanii* (ATCC 50492) (control strain of CPE). Images are representative of experiments performed in triplicate.

The in vitro cytopathic effects (CPEs) of the *Acanthamoeba* isolates were assessed using the crystal-violet staining method and evaluated based on the degree of monolayer destruction. Figure 3 shows the vital disruptive effect of the crystal-violet stain over the HeLa cell monolayer after 24 h of incubation of the amoebae. Evidently, the monolayer was disrupted by the trophozoites, which were also attached to the plate in the spaces previously occupied by HeLa cells or between the attached cells.

After 24 h, only four T4 isolates (CL5, CL54, CL149 and TB5-B27) incubated with the HeLa cell monolayer were classified as having CPEs, with over 50–100% (+++) of the cell damaged. Additionally, well plates containing more than 50% trophozoite lysates from potentially pathogenic *Acanthamoeba* isolates showed the presence of holes, suggesting the supporting role of extracellular protease in *Acanthamoeba* isolates in the epithelial-cell disaggregation. Furthermore, 23 isolates, including SKA5-SK35 (T3 genotype), were categorized as having CPEs with 10–50% (++) cell damage, while 13 isolates were group as having CPEs with less than 10% (+) of destruction of the cell monolayer (Table 3). In contrast, the HeLa cell monolayer incubated with *A. castellanii* (ATCC 50492), which represents the positive control sample, showed 56% alteration throughout the CPE assay. The only sample that showed no alteration in the CPE assay was the monolayer without amoeba, which represented the negative control sample.

### 3.5. Trophozoite Lysate Demonstrated In Vitro Cytopathogenicity against HeLa Cell Monolayer

As shown in Table 3, the LDH assay showed that potentially pathogenic *Acanthamoeba* isolates produced a significant in vitro cytopathogenicity level in the HeLa cell monolayer after 24 h of incubation. Three T4 isolates (CL5, CL54 and CL149) and a single T3 isolate (SKA5-SK35) were highly toxic, as indicated by the LDH release of more than 40%. On the contrary, a total of 24 *Acanthamoeba* isolates (including TB5-B27) demonstrated LDH releases of around 10–40%, implying a low-to-intermediate cytotoxicity, while only 12 isolates recorded an LDH release of less than 10%, signifying the nontoxic properties of the isolates.

## 4. Discussion

Amoebic keratitis has been identified as an acute ocular amoebic infection in which the application of contact lenses contributes a key risk factor [39]. This is because they induce changes in the epithelial layer and decrease the corneal epithelial resistance to microbial invasion [6]. Currently, information regarding the biological and cytopathogenic mechanisms between the varying *Acanthamoeba* genotypes and their relationship with the virulence of each strain is limited and not yet established. Thus, the findings in this study showed that twelve T4 genotypes and singular T3 and T15 genotypes, isolated from both contact lens paraphernalia and an environmental water source in Malaysia, were thermotolerant and osmotolerant, which represent indirect pathogenic factors [8]. Interestingly, the findings correspond to the high thermo- and osmotolerance of 16 *Acanthamoeba* T4 isolates collected from the ocular surface of contact lens wearers [40]. In fact, the predominant T4 genotype isolated from the environment displayed strong thermotolerance and osmotolerance [36]. Additionally, the T3 and T15 genotypes showed a higher capability to grow under both harsh environments compared to that reported in a previous study [41]. In another study conducted in Brazil, it was revealed that the T15 genotype showed a highly pathogenic potential, although it was characterized as a non-keratitis-causing strain [37]. The genotype was reported to retain a longer bacterial symbiont relationship compared to other *Acanthamoeba* genotypes. It is crucial to emphasize that the amoeba–endosymbiont interaction could play a role in the pathogenic mechanism of the *Acanthamoeba* species [1]. Furthermore, thermotolerance refers to the persistence ability of the amoeba against normal body temperatures or periodic fever in the host. In fact, the growth of amoebae at temperatures higher than 40 °C is directly linked to their ability to cause in vitro cellular disruption [42]. On the other hand, the growth of amoebae under high mannitol concentrations has been assumed to correspond with the resistance level under high osmotic pressure, which the amoebae could experience when behaving as parasites in the corneal epithelium [1]. Nevertheless, both characteristics are insufficient to define the pathogenicity of a particular amoeba, despite their adaptive advantages when parasitizing a host. In other words, certain *Acanthamoeba* species could possess high thermotolerance but may be nonpathogenic [43]. The pathogenicity level of an amoeba that infects the human cornea could not be fully defined based on these physiological properties because the temperature of the cornea is approximately between 32 and 35 °C. Therefore, the amoeba could still colonize the host, although the amoeba is unable to grow at temperatures over 37 °C [44]. Apart from that, it is essential to highlight that dissimilar isolates from the same genotype could exhibit various pathogenic potentials given their ability to adapt to the nature of their origin. Moreover, the infectious capability of the strains under harsh environmental stress may be linked to several factors, such as the release of heat-shock proteins (HSP70) [45].

Although proteases play an essential role in the biological pathogenesis of *Acanthamoeba*, knowledge regarding these enzymes is still scarce [6]. To date, very few studies on extracellular proteases have been conducted using a limited number of *Acanthamoeba* isolates from different species and genotypes. In this study, the secretion of extracellular serine proteases was identified in all *Acanthamoeba* isolated from contact lens paraphernalia and environmental origins. The migration pattern, with molecular weights of 45–230 kDa, confirmed the predominant type in the repertoire of proteolytic enzymes described for *Acanthamoeba* [46]. The findings were similar to those reported using other *Acanthamoeba* in past studies, including clinical isolates [6,36]. Remarkably, the present study detected the presence of a serine protease with a molecular weight of 133 kDa in three T4 isolates from the contact lens paraphernalia samples. The finding was supported by another previous study that also detected a serine protease, with a similar molecular weight of 133 kDa, from the clinical isolates, which triggered a cytotoxic effect in both human and hamster corneal epithelial cells [19]. In a recent study, a 133 kDa serine protease, termed MIP133, was identified as a vital constituent of the pathogenic cascade of the *Acanthamoeba* pathogenesis. The MIP133 serine protease was found to trigger the decomposition of keratocytes, iris ciliary body cells, retinal pigment epithelial cells, corneal epithelial cells and corneal endothelial cells, as well as induce apoptosis in macrophage-like cells [17]. It is also important to note that this study detected the presence of protease activity with a molecular weight of 107 kDa, which has been specifically demonstrated in *Acanthamoeba* isolates from clinical samples [18]. Thus, it is expected that the overexpression of the 107 kDa protease in pathogenic *Acanthamoeba* isolates could be utilized as a diagnostic tool to differentiate *Acanthamoeba* species. Earlier reports have considered the use of serine proteases as pathogenicity indicators, with proven action not only on proteases in plasminogen activation but also on collagen and fibronectin degradation [6]. Previously, Omana-Molina et al. [47] verified the functional role of proteases in tissue invasion, which highlighted their involvement in extracellular matrix digestion but not in the direct cellular lysis process. It is also crucial to note that the enzymatic activities have been reported particularly in clinically isolated *Acanthamoeba* from keratitis or encephalitis [24]. For environmental isolates, a 56 kDa proteolytic enzyme, which was similar to that in the present study (genotype T3), has been identified from a contact-lens-wearing keratitis patient in Spain [48]. Acknowledging their necessary presence for the eventual facultative parasitism in amoeba, Dudley et al. [49] proposed the role of serine proteases in nutrition and encystment/excystment. Furthermore, this study detected the presence of proteolytic enzymes from trophozoite lysates that were inhibited by PMSF at 252, 162, 152, 100 and 80 kDa. The results correspond with the role of serine proteases in pathogenesis and conforms to the findings of Alfieri et al. [33], whose study also used PMSF as a serine protease inhibitor. They deduced that the 100, 75 and 47 kDa bands produced by *Acanthamoeba polyphaga* lysate from human keratitis were of serine protease in nature. In addition, Tawfeek et al. [12] discovered that the proteolytic activity of pathogenic *Acanthamoeba* strains with a molecular weight of 100 kDa were serine protease in nature, as it was inhibited by PMSF.

Besides the production of extracellular proteases, the pathogenicity of *Acanthamoeba* is also influenced by cell surface adhesion. The simultaneous adhesion and cytotoxicity assay in this study showed the distinct variations between pathogenic and nonpathogenic *Acanthamoeba* isolates in terms of adherence to the HeLa cell monolayer, which also implies the adhesion process as a vital primary step in the pathogenesis of *Acanthamoeba*. HeLa are the most common cells used to investigate the effects of *Acanthamoeba* towards cell lines [48,50]. HeLa cells also exhibit epithelial morphology and can grow adherently. The T3 and T4 genotypes from both isolates recorded a strong adherence to the cell monolayers with an adhesion rate of up to 90–98.5%. Predictably, the amoeba isolates highly influenced the HeLa cell monolayer, which was also reported in previous studies using identical cell line cultures [50,51]. For example, Martin-Navarro et al. [50] reported an exceptional rate of adhesion of more than 95.8% by T3, T4 and T15 *Acanthamoeba* genotypes onto the HeLa cell line. However, the adhesion rate varied between different *Acanthamoeba* genotypes using Madin–Darby canine kidney (MDCK)-cell monolayers [52,53]. The inconsistent adherence level to the MDCK cell line could be due to the conformational difference in the mannose-binding protein (MBP) between the studied strains and the contact-dependent mechanism [54,55]. Given that the MBP is characterized as a typical cell-surface transmembrane receptor protein, future studies should consider addressing the differences in the MBP among *Acanthamoeba* isolates. 

The HeLa cell line was applied in this study as a cytolytic model to evaluate the potential cytopathic effects (CPEs) and in vitro cytopathogenicity of the *Acanthamoeba* isolates [48,56]. Meanwhile, the crystal-violet staining enabled the viewing of the damage to the monolayer following the addition of CL5, CL54, CL149, TB5-B2 and SKA5-SK35 or the *A. castellanii* strain ATCC 50492 (control strain) after 24 h of incubation. Based on the results, the disaggregation of the monolayer by amoeba trophozoites attached to the plate in the spaces previously occupied by the cells, or between the attached cells, was observed as the main effect in the assay. The cytotoxicity levels of the same isolates and the control strain were associated with the degree of cellular damage, as observed in the crystal-violet assay (except for TB5-B27) when the LDH-release assay was used. This finding could be explained by assuming that the CPE for the TB5-B27 isolate was due to the cell-line lysis instead of the disruption of the cell monolayers. In accordance with the findings in the present study, Martin-Navarro et al. [50] reported cytotoxicity percentages of 60–70% for *Acanthamoeba* strain Neff and 55–75% for *Acanthamoeba* strain CLC-16. In a previous study, Castro-Artavia et al. [36] employed MDCK cell lines and reported a cytotoxicity percentage of 14.4–24% for the T4 genotype (*Acanthamoeba* strains DU12 and CSUT7) and 38.8% for the T3 genotype (*Acanthamoeba* strain CLC-16). Comparatively, the cytotoxic percentages were substantially lower than those achieved in the present study, which could be associated with the type of cell line used in the interaction. Regardless of the results in the literature, the findings in the present study suggest that the environmental isolates, particularly TB5-B27 (T4 genotype) and SKA5-SK35 (T3 genotype), recorded an in vitro CPE and were able to significantly damage the cell monolayer. It is also essential to emphasize that the CPE assay was conducted after the cultures were subjected to 10 passages, which lasted for a maximum of five weeks (data not shown). Despite the relatively short monoxenic maintenance time, the attenuation in the CPE ability could not be dismissed. Nevertheless, the expression of the CPE, even at low levels, indicates the pathogenicity that was considered in the analysis.

## 5. Conclusions

Overall, the present study demonstrated the pathogenic potential of three T4 (CL5, CL54 and CL149) and one T3 (SKA5-SK35) *Acanthamoeba* genotypes isolated from contact lens paraphernalia and a water source, respectively. The amoebic pathogenicity could represent an intrinsic physiological property of a specific species and *Acanthamoeba* infection could correlate with amoebic features, such as growth temperature, proteolytic activity, adhesion and in vitro cytopathogenicity. In addition, this study suggests a considerable variation in the response of *Acanthamoeba* species of the same genotype to physiological and biochemical pathogenicity indicators, which complicates the classification process. Further detailed analyses would also determine the possibility of include subclades with potentially varying degrees of virulence and pathogenicity. Moreover, the high release of LDH of more than 40% by the T3 genotype isolated from the environmental samples underlines the significant potential to trigger infections. Hence, the results demonstrate the urgent need to reassess the functional role of other *Acanthamoeba* genotypes that were previously classified as nonpathogenic isolates in the production of amoebic keratitis and other nonkeratitis infections.

## Figures and Tables

**Table 1:** Origin, genotype and physiological characteristics of *Acanthamoeba* isolates used in this study.

Origin	Isolate	Genotype	Growth at		Growth at		Pathogenic Potential
			37 °C	42 °C	0.5 M	1 M	
Contact Lens	CL5	T4	+++	+	+++	+++	High
Paraphernalia	CL45	T4	+++	−	+++	−	Low
	CL54	T4	+++	+++	+++	+++	High
	CL107	T4	+++	−	+++	−	Low
	CL118	T4	+++	++	+++	+++	High
	CL126	T4	+++	++	+++	+++	High
	CL149	T4	+++	+	+++	+++	High
	CL173	T4	+++	−	+++	−	Low
	CL176	T4	+++	−	+++	−	Low
	CL177	T4	−	−	+++	−	Non
Environmental							
Hot Spring	SA1-SLG1	T4	+++	++	++	−	Low
	SD1-SLG9	T4	+++	++	+	++	High
	UTA4-HT14	T4	+++	+	++	+	High
	UTA5-HT15	T4	+++	−	++	−	Low
	BTGB4-BTG29	T4	+++	++	+	−	Low
	SKA1-SK31	T4	+++	−	++	−	Low
	GB1-GDK43	T4	+++	++	++	−	Low
	GB2-GDK44	T4	+++	++	++	+	High
	GB4-GDK46	T4	+++	−	++	−	Low
	GC3-GDK49	T4	+++	+	++	+	High
Beach	M4−B4	T4	+	−	+	+	Low
	M5-B5	T4	+++	+	++	+++	High
	PD2-B11	T4	+	−	−	−	Non
	M9-B9	T4	+	−	−	−	Non
	PD3-B12	T4	+	−	+	+	Low
	L1-B18	T4	+++	+	+	++	High
	L8-B22	T4	+	−	+	+	Low
	TB5-B27	T4	++	+	++	++	High
	TC1-B30	T4	++	−	−	+	Low
	TC4-B33	T4	+	−	+	+	Low
Non-T4	UTB3-HT18	T15	+++	−	++	−	Low
	UTC1-HT20	T15	+++	+	+	+	High
	BTGA3-BTG23	T15	+++	+	−	−	Low
	SKA3-SK33	T3	+++	++	++	−	Low
	SKA5-SK35	T3	+++	+	+	+	High
	BTGB5-BTG30	T17	+++	++	++	−	Low
	PD5-B14	T5	+	−	+	+	Low
	TC7-B34	T11	+	−	+	+	Low
	M8-B8	T18	++	−	+	+	Low
	L6-B21	T20	−	−	−	−	Non
Reference							
ATCC 50492	*A. castellanii*	T4	+++	++	+++	++	High

− = 0; + = 1–15; ++ = 16–30; +++ = >30.

**Table 2:** Protease profile (proteases number and molecular weights) of trophozoite lysate samples prepared from different isolates.

No.	Isolate	Genotype	Total Protease No. (MWs (kDa))	Serine Protease No. (MWs (kDa))	Unclassified Protease No. (MWs (kDa))
1	CL5	T4	4 (230, 148, 133, 95)	4 (230, 148, 133, 95)	-
2	CL45	T4	2 (230, 67)	2 (230, 67)	-
3	CL54	T4	4 (230, 122, 107, 85)	4 (230, 122, 107, 85)	-
4	CL107	T4	4 (230, 122, 100, 85)	3 (230, 122, 85)	1 (100)
5	CL118	T4	1 (230)	1 (230)	-
6	CL126	T4	4 (228, 133, 120, 92)	4 (228, 133, 120, 92)	-
7	CL149	T4	5 (226, 133, 120, 92, 75)	5 (226, 133, 120, 92, 75)	-
8	CL173	T4	3 (228,120, 92)	3 (228,120, 92)	-
9	CL176	T4	4 (228, 148, 120, 100)	3 (228, 148, 120)	1 (100)
10	CL177	T4	4 (228, 122, 100, 92)	3 (228, 122, 92)	1 (100)
11	SA1-SLG1	T4	2 (100, 84)	2 (100, 84)	-
12	SD1-SLG9	T4	2 (75, 62)	2 (75, 62)	-
13	UTA4-HT14	T4	1 (210)	1 (210)	-
14	UTA5-HT15	T4	4 (122, 85, 66, 52)	4 (122, 85, 66, 52)	-
15	BTGB4-BTG29	T4	2 (220, 192)	2 (220, 192)	-
16	SKA1-SK31	T4	1 (124)	1 (124)	-
17	GB1-GDK43	T4	2 (120, 70)	2 (120, 70)	-
18	GB2-GDK44	T4	2 (252, 193)	1 (193)	1 (252)
19	GB4-GDK46	T4	3 (230, 176, 148)	3 (230, 176, 148)	-
20	GC3-GDK49	T4	1 (55)	1 (55)	-
21	M4-B4	T4	4 (162, 85, 70, 55)	4 (162, 85, 70, 55)	-
22	M5-B5	T4	1 (85)	1 (85)	-
23	PD2-B11	T4	2 (162, 70)	1 (70)	1 (162)
24	M9-B9	T4	2 (85, 55)	2 (85, 55)	-
25	PD3-B12	T4	4 (165, 157, 70, 75)	4 (165, 157, 70, 75)	-
26	L1-B18	T4	4 (160, 92, 85, 45)	4 (160, 92, 85, 45)	-
27	L8-B22	T4	2 (160, 85)	2 (160, 85)	-
28	TB5-B27	T4	2 (120, 97)	2 (120, 97)	
29	TC1-B30	T4	6 (177, 162, 100, 85, 70, 55)	5 (177,100, 85, 70, 55)	1 (162)
30	TC4-B33	T4	7 (177, 162, 152, 100, 85, 70, 55)	5 (177, 100, 85, 70, 55)	2 (162, 152)
31	UTB3-HT18	T15	2 (152, 55)	2 (152, 55)	-
32	UTC1-HT20	T15	3 (178, 70, 47)	3 (178, 70, 47)	-
33	BTGA3-BTG23	T15	2 (85, 64)	2 (85, 64)	-
34	SKA3-SK33	T3	3 (148, 75, 56)	3 (148, 75, 56)	-
35	SKA5-SK35	T3	5 (148, 97, 75, 64, 56)	5 (148, 97, 75, 64, 56)	-
36	BTGB5-BTG30	T17	5 (160, 122, 80, 64, 55)	4 (160, 122, 64, 55)	1 (80)
37	PD5-B14	T5	1 (157)	1 (157)	-
38	TC7-B34	T11	3 (130, 97, 62)	3 (130, 97, 62)	-
39	M8-B8	T18	4 (148,97,70,62)	4 (148,97,70,62)	-
40	L6-B21	T20	3 (148, 67, 49)	3 (148, 67, 49)	-
41	*A. castellanii* (ATCC 50492)	T4	5 (148, 133, 92, 64, 58)	5 (148, 133, 92, 64, 58)	-

MW = Molecular weight.

**Table 3:** Percentages of adhesion, cytopathic effects and cytopathogenicity (LDH release) at 24 h after inoculation of the different isolates of *Acanthamoeba* with HeLa cell line.

Origin	Isolate	Genotype	% of Adhesion	Cytopathic Effect		Mean Cytotoxicity Level LDH (%)
				%	Grade	
Contact Lens	CL5	T4	96.6 ± 0.6	59.4 ± 1.0	+++	50.9 ± 2.8
Paraphernalia	CL45	T4	94.5 ± 0.4	36.3 ± 1.3	++	28.3 ± 0.6
	CL54	T4	95.9 ± 1.0	50.8 ± 1.7	+++	56.3 ± 0.3
	CL107	T4	95.1 ± 0.3	38.8 ± 2.2	++	27.2 ± 1.0
	CL118	T4	96.5 ± 0.6	35.4 ± 2.8	++	21.3 ± 0.3
	CL126	T4	85.9 ± 1.4	24.7 ± 1.7	++	15.5 ± 1.7
	CL149	T4	98.5 ± 0.2	55.0 ± 1.6	+++	60.6 ± 2.5
	CL173	T4	85.9 ± 1.3	9.7 ± 0.7	+	12.9 ± 2.7
	CL176	T4	80.2 ± 1.6	9.0 ± 2.2	+	9.2 ± 0.8
	CL177	T4	78.9 ± 1.8	3.5 ± 0.3	+	9.0 ± 1.2
Environmental						
Hot Spring	SD1-SLG9	T4	87.8 ± 2.5	21.4 ± 0.5	++	22.8 ± 0.2
	UTA4-HT14	T4	90.5 ± 0.6	41.7 ± 1.5	++	29.3 ± 1.8
	GB2-GDK44	T4	85.7 ± 1.0	14.2 ± 2.0	++	11.2 ± 0.4
	GC3-GDK49	T4	85.6 ± 0.5	30.1 ± 0.7	++	20.1 ± 0.4
	UTA5-HT15	T4	79.2 ± 1.6	9.5 ± 1.8	+	10.2 ± 0.6
	BTGB4-BTG29	T4	75.3 ± 0.4	11.8 ± 1.0	++	14.3 ± 1.9
	SKA1-SK31	T4	85.4 ± 0.0	20.3 ± 0.9	++	16.2 ± 0.9
	GB1-GDK43	T4	85.3 ± 0.6	20.4 ± 0.6	++	12.8 ± 0.8
	SA1-SLG1	T4	84.8 ± 0.2	10.7 ± 1.1	+	10.7 ± 1.5
	GB4-GDK46	T4	74.7 ± 1.1	9.6 ± 1.4	+	9.9 ± 1.4
Beach	M4-B4	T4	85.5 ± 0.9	14.7 ± 1.7	++	7.1 ± 0.1
	M5-B5	T4	86.8 ± 1.0	26.6 ± 1.3	++	16.8 ± 0.6
	PD2-B11	T4	78.9 ± 1.4	9.2 ± 1.9	+	4.1 ± 0.4
	M9-B9	T4	77.7 ± 0.7	8.1 ± 2.2	+	8.6 ± 0.6
	PD3-B12	T4	62.4 ± 2.3	9.8 ± 2.3	+	3.3 ± 1.5
	L1-B18	T4	89.7 ± 0.3	27.3 ± 1.5	++	23.7 ± 1.0
	L8-B22	T4	78.0 ± 1.6	13.7 ± 1.7	++	9.6 ± 1.2
	TB5-B27	T4	90.8 ± 1.2	55.2 ± 0.6	+++	32.6 ± 2.5
	TC1-B30	T4	72.8 ± 1.0	17.8 ± 1.8	++	10.8 ± 0.8
	TC4-B33	T4	69.3 ± 1.1	8.8 ± 2.3	+	4.4 ± 1.0
Non-T4	UTB3-HT18	T15	83.1 ± 0.5	9.3 ± 1.6	+	12.9 ± 1.5
	UTC1-HT20	T15	87.8 ± 2.0	30.5 ± 0.9	++	33.7 ± 1.9
	BTGA3-BTG23	T15	78.3 ± 1.0	14.4 ± 1.3	++	11.6 ± 1.0
	SKA3-SK33	T3	86.5 ± 1.5	38.7 ± 1.5	++	32.4 ± 0.6
	SKA5-SK35	T3	96.5 ± 0.7	44.9 ± 0.6	++	50.2 ± 0.1
	BTGB5-BTG30	T17	85.2 ± 0.8	15.6 ± 1.0	++	10.4 ± 0.3
	PD5-B14	T5	85.7 ± 1.4	12.3 ± 1.2	++	15.0 ± 0.6
	TC7-B34	T11	57.5 ± 2.7	9.9 ± 2.2	+	2.1 ± 0.5
	M8-B8	T18	69.5 ± 1.8	4.9 ± 1.1	+	9.8 ± 0.7
	L6-B21	T20	72.3 ± 2.3	0.7 ± 0.2	−	9.1 ± 1.3
Reference						
ATCC 50492	*A. castellanii*	T4	98.7 ± 0.2	56.0 ± 1.6	+++	61.2 ± 2.3

## Data Availability

Data supporting the findings of this study are available in the main text.
